# Promising Extracellular Matrix (ECM) Enzymes: Collagenase II and Chondroitinase as Non-surgical Interventions in Correcting Ear Deformities

**DOI:** 10.7759/cureus.98363

**Published:** 2025-12-03

**Authors:** Chenxu Chen, Xiaohui Su, Meiling Weng, Hailing Lai, Jiong Ye, Houbing Zheng, Meishui Wang, Chen Lei

**Affiliations:** 1 Plastic Surgery, The First Affiliated Hospital, Fujian Medical University, Fujian, CHN; 2 National Regional Medical Centre, Binhai Campus of the First Affiliated Hospital, Fujian Medical University, Fujian, CHN

**Keywords:** cartilage reshaping, chondroitinase, collagenase ii, ear deformities, ecm enzymes

## Abstract

This hypothesis addresses the challenges of non-surgical treatment for congenital auricular deformities by exploring the feasibility of using digestive enzymes, such as collagenase and chondroitinase (ChSase), for cartilage remodeling. Congenital auricular deformity is a common external malformation, and some cases require clinical intervention for correction. Although surgical treatment is well-established, it carries associated risks, making early non-surgical intervention particularly important. Current non-surgical treatments rely heavily on the neonatal cartilage's pliability but are limited by a narrow window of opportunity dictated by hormonal influences after birth. To overcome this barrier, we propose a hypothesis that local injection of digestive enzymes can modulate the mechanical properties of the cartilage matrix, thereby promoting cartilage reshaping. By leveraging enzymes like collagenase and ChSase, selective degradation of cartilage matrix elements is achieved, inducing plasticity while preserving cellular viability. This strategy has the potential to extend the window of opportunity for cartilage reshaping and provides a novel theoretical basis for early non-surgical correction of congenital auricular deformities. We conclude that although this hypothesis may appear overly simplistic, it is feasible and merits further investigation.

## Introduction

Congenital auricular anomaly is one of the most common deformities in our country, China, with its incidence rate varying from 20.9% to 20.9%-57.5% [1]. It can be briefly categorized into deformity and malformation, including lop ear, cup ear, Stahl’s ear, prominent ear, etc [2,3]. Deformities are primarily caused by perinatal external forces or abnormal development of auricular muscles, characterized by morphological changes of the auricle while maintaining a relatively normal cartilaginous framework. In contrast, malformations result from impaired morphogenesis of the first and second branchial arches during embryonic development and are often associated with substantial deficits in skin and cartilage tissue [4]. Notably, one-third of the neonatal auricular deformities can be self-corrected within seven days after birth, when auricular cartilage still retains its plasticity. However, 10% to 15% of cases still require clinical intervention [5]. Since the auricle is a part of the facial aesthetic area, anomalies here may bring psychological and social stress to the patients. A previous study indicated that the incidence rate of psychological social problems was higher in children with auricular anomalies than in those without [6]. Therefore, early correction is important to prevent significant psychological impacts. Though operative repair is currently recognized as the optimal treatment for ear malformation, the underlying risks of anesthetic and postoperative complications underscore the importance of non-surgical correction [7].

Temporary splinting using surgical tapes or Reston foam was first proposed by Matsuo et al. and Kurozumi et al. in the 1980s as a method to correct congenital ear deformities [8,9]. Since then, various splinting correction techniques have been described [10]. These early attempts laid the foundation for the basic principles of ear molding. The technique was later standardized with the invention of EarWell® by Byrd et al., a non-invasive device specifically designed for the correction of ear deformities [5]. However, the narrow window of opportunity has greatly hindered the broader application of the non-surgical approaches. The feasibility of neonatal ear molding is mainly ascribed to the high concentration of proteoglycans, including hyaluronic acid, whose levels are elevated by the circulating maternal estrogen [3,5,8]. The auricles then gradually become firm following the rapid decline of circulating estradiol within 72 hours after birth [11]. Accordingly, the timing of initial non-surgical correction is critical for success, and the outcomes are highly correlated to how early the intervention is started [1,12]. Therefore, we hypothesize that replicating the early neonatal pliability of auricular cartilage through biomechanical modulation may offer a potential breakthrough for non-surgical approaches. 

Various attempts were made to manipulate cartilage mechanical properties by local prescription of estrogen or hyaluronic acid filler in rabbit models [13-16]. Nevertheless, these attempts lacked physical and histological evidence to support their long-term efficacy. Moreover, the clinical application of hormones in children should be approached with caution, especially considering their potential effects on the endocrine system and overall growth [17].

## Technical report

Enzyme-mediated ear molding strategies

The enzyme-mediated approach is another promising way for the mechanical modulation of cartilage. A previous study has shown that the treatment of collagenase enhanced the plasticity of cartilage strips [18]. Gandya et al. further demonstrated that local injection of collagenase effectively reduced the mechanical strength of auricular cartilage in rabbits, enabling reshaping after bending and fixation [19]. These promising findings point to a novel enzymatic strategy for non-surgical ear molding by precisely manipulating the enzymatic degradation dynamics of key extracellular matrix (ECM) components. Based on the above, this article aims to analyze the effects of various enzymes on cartilage components and to demonstrate chondroplastic potential through enzyme-mediated manipulation of cartilage mechanical strength.

Components of the cartilage matrix and its mechanical strength

Compositions of the Cartilage Matrix and Their Function

Cartilage contains chondrocytes and their ECM, which is composed of water, collagen, proteoglycans, and non-collagenous glycoproteins [20].

Water accounts for approximately 75% to 80% of the wet weight of cartilage. It plays a crucial role in maintaining the elasticity, compressive resistance, and molecular transport [21]. Collagen is a major protein secreted by connective tissue cells and is essential for maintaining the structural and biological integrity of various tissues and organs, including bone, skin, tendons, blood vessels, and cartilage [22]. Among the different types of collagen, type II collagen is the predominant form in the cartilage ECM. It forms a fibrous network that constitutes one of the two main structural elements of the ECM, playing a vital role in preserving the architecture and function of cartilage [23]. 

Proteoglycans are composed of a core protein covalently attached to one or more glycosaminoglycan (GAG) chains [20]. The major GAGs include chondroitin sulfate, dermatan sulfate, heparan sulfate, hyaluronic acid (HA), and keratan sulfate [24]. Among them, aggrecan, primarily composed of chondroitin sulfate and keratan sulfate, is the most abundant proteoglycan in cartilage. Due to their high negative charge, proteoglycans can attract and retain large amounts of water, forming a gel-like matrix that provides high osmotic pressure and elasticity, which are essential for the load-bearing and compressive properties of cartilage [25]. Non-collagenous glycoproteins, including fibronectin, laminin, and mucins, also contribute to the ECM [26]. These molecules are involved in cell-matrix interactions, cell adhesion, and signaling regulation, thereby playing a crucial role in the growth, differentiation, and maintenance of chondrocytes [26].

In addition to serving as a mechanical scaffold, the cartilage matrix also acts as a reservoir for various growth factors and cytokines. These bioactive molecules regulate matrix metabolism, cellular activity, and tissue repair and are involved in both physiological homeostasis and pathological processes such as osteoarthritis [20].

Mechanical Factors of the Cartilage

The mechanical strength of the cartilage matrix primarily derives from its highly organized hierarchical structure [27], the core of which consists of the synergistic interaction between the collagen fiber network and the proteoglycan-water complex.

Collagen II-rich fibrils in cartilage serve as a framework that can resist strong tension, and the fibrillar network contains swelling tension. These provide the cartilage tissue with elasticity and stiffness to tolerate compressive and tensile loading [20,28]. Negatively charged proteoglycans can attract osmotically active Na+. As a consequence, water is drawn into the cartilaginous matrix, forming a fully hydrated gel-like structure with a low concentration [20]. This structure absorbs compressive forces through the redistribution of water molecules, thereby preventing tissue collapse and providing resilience [25]. This so-called "rebar-concrete" architecture, where collagen fibers serve as the "rebar" and the proteoglycan-water complex functions as the "concrete", endows cartilage with its unique viscoelastic properties.

Mechanism of enzymatic molding: a dynamic balance between degradation and regeneration

Enzyme-mediated cartilage molding technique can effectively reduce cartilage stiffness; however, it poses a long-term risk of structural instability due to the unidirectional degradation of the ECM. This suggests the need for the establishment of a dynamic degradation-regeneration system to preserve the structural and functional integrity of the ECM. Interestingly, chondrocytes are not only responsible for generating the cartilage ECM but also for maintaining its homeostasis. In addition to synthesizing key matrix components such as proteoglycans, they actively participate in matrix degradation, thereby playing a crucial role in preserving tissue homeostasis through the balanced regulation of anabolic and catabolic processes [29]. This suggests that chondrocytes have the potential to continuously generate most of the ECM components.

Yanaga et al. successfully regenerated solid cartilage tissue with newly generated matrix by subcutaneously implanting auricular chondrocytes, which were isolated from native cartilage through collagenase digestion and cultured in vitro for a period of time [30]. The regenerated cartilage exhibited histological consistency with native auricular cartilage and was later sculpted for ear reconstruction. Notably, this spontaneous regeneration of cartilage matrix by chondrocytes effectively reversed the in vitro enzymatic digestion process. This is essential for the reshaping process of our enzymatic ear molding strategy. In our strategy, since the enzymatic degradation process occurs subcutaneously at where the auricle is naturally at, the digested chondrocyte can be assumed to be an in-situ cell reimplantation. We hypothesize that the natural local microenvironment is more conducive to the spontaneous regeneration of cartilage matrix by chondrocytes.

Based on the above, we propose the following hypothesis: local injection of carefully selected and controlled matrix-degrading enzymes can modulate the mechanical properties of the auricle and induce the spontaneous regeneration of ECM by the enzyme-digested chondrocytes in situ.

The enzymatic digestion occurs beneath the auricular skin, precisely at the original site of the cartilage tissue. This implies that the digested chondrocytes effectively undergo in situ reimplantation. Our team believes that the unchanged microenvironment during the experiment is more conducive to the spontaneous regeneration of cartilage matrix by chondrocytes.

Therefore, we propose that local injection of collagenase selectively digests the auricular cartilage matrix, altering the mechanical strength of the auricular cartilage and conferring plasticity. Subsequently, the chondrocytes regenerate new matrix along the fixed shape, thus achieving reshaping of the auricular cartilage.

Comparative analysis and optimization strategies of digestive enzymes

Collagenase

Collagenase is a protease that specifically degrades collagen fibers. Since collagen fibers constitute the primary source of mechanical strength in the cartilage matrix, their selective degradation substantially reduces cartilage rigidity [20,28]. *Clostridium histolyticum* collagenase (trade name Xiaflex®) is FDA-approved and widely used for fibroproliferative disorders [31,32]. This enzyme does not degrade type IV collagen, the main constituent of basement membranes, blood vessel walls, and perineurium [27,33]. Consequently, Xiaflex® can be safely applied even in anatomical regions densely populated with nerves, blood vessels, and tendons [31,32]. Nevertheless, subcutaneous administration has been associated with injection-site pain, edema, allergic reactions, and hemorrhagic complications such as hematoma and ecchymosis [34]. Most adverse events are mild and self-limiting, typically causing only a delay rather than termination of therapy. However, further preclinical and clinical monitoring is required to confirm long-term safety.

*Chondroitinase (ChSase*)

Chondroitin sulfate lyase or ChSase is a family of enzymes that depolymerize chondroitin sulfate and HA into unsaturated disaccharides and oligosaccharides [35]. By degrading GAGs, ChSase disrupts the proteoglycan-water complex, dehydrates the cartilage matrix, and markedly reduces its mechanical strength [25]. Clinically, ChSase ABC has been used to digest glial scar tissue and enhance neural regeneration after central nervous system injury [36,37]. It has also been administered intradiscally for lumbar disc herniation [38]. No definitive clinical complications have been reported to date; however, non-specific degradation of dermal HA may pose a risk of tissue injury [39]. Moreover, native ChSase exhibits poor thermostability, with enzymatic activity declining significantly within three days at 37℃ and within one day at 39℃, thereby severely limiting its practical applications [40].

*Hyaluronidase (HAase*)

HAase or hyaluronate lyase is a glycosidase that primarily hydrolyzes HA [41]. Like ChSase, HAase is part of the GAG-degrading enzyme family and shares both structural and functional similarities. Based on catalytic mechanisms, HAase variants include HA lyases, hyaluronoglucosidases, and hyaluronoglucuronidases [42]. HAase-mediated HA degradation dissolves the proteoglycan-water complex, leading to cartilage dehydration [25]. Commercial bovine testicular HAase (BTH) and recombinant human PH20 (rHuPH20) are widely used as adjuvants to enhance drug diffusion (e.g., local anesthetics, immunoglobulins) [41] and as standard therapies for complications from HA-based dermal fillers [43,44]. Limitations include dermal HA degradation that may exacerbate local inflammation and the immunogenicity associated with animal-derived BTH, which can trigger allergic reactions and neutralizing antibodies that impair efficacy [45]. Although rHuPH20 significantly reduces allergenicity, it still faces constraints due to a short half-life and rapid degradation, limiting its clinical application [46].

Trypsin

Trypsin is a serine protease that specifically cleaves peptide bonds at the carboxyl side of arginine (Arg) and lysine (Lys) residues, yielding soluble short peptides [47]. It can be combined with other enzymes to digest cartilage matrix components for chondrocyte isolation or used to modulate the cartilage matrix's mechanical strength. However, enzymatic remodeling must maintain a dynamic balance between degradation and regeneration. Due to its broad non-specificity, trypsin digests most native proteins. This may result in collateral damage to perichondrial tissues, trigger severe inflammation that impedes regeneration, and cause the cleavage of cell-surface receptors or membrane proteins, ultimately leading to cellular dysfunction [48]. Consequently, trypsin is unsuitable for enzymatic cartilage-shaping strategies. 

## Discussion

Targeted enzymatic sculpting precisely modulates the activity of collagenase or ChSase to selectively degrade key ECM components of cartilage, thereby reducing its mechanical strength and enabling plastic deformation under applied force. This approach circumvents the narrow, neonatal estrogen-sensitive time window seen with traditional non-surgical treatments, providing new therapeutic opportunities for patients who miss the optimal intervention period.

Empirical data indicate that, in cartilage blocks of identical size, ChSase induces a homogeneous, full-thickness loss of safranin-O staining. In contrast, collagenase produces a gradual, inside-out fading with a frayed periphery [49]. This suggests collagenase digestion is a gradual, externally initiated collapse, whereas ChSase rapidly permeates the entire cartilage thickness. Therefore, ChSase is likely to alter cartilage mechanical strength more rapidly than collagenase. Overall, the optimal hierarchy of digestive enzymes for cartilage remodeling is ChSase > collagenase > HAase.

Clinical translation must still overcome critical challenges, particularly ensuring a controlled balance between matrix degradation and regeneration by endogenous chondrocytes to restore matrix integrity and prevent long-term structural instability. If such an equilibrium can be achieved, this technique could evolve into a minimally invasive and precise cartilage reshaping tool. It would significantly broaden the indications and the window of opportunity for non-surgical correction of auricular deformities while also reducing surgical burden and psychological risk for pediatric patients. Gandy et al. [19] have previously conducted animal studies of cartilage reshaping on excised rabbit ears, achieving effective shaping results through enzymatic injections. In future studies, this technique will be further explored in live animal models.

Nevertheless, the potential effects on pediatric patients should be carefully considered, as most recipients are in active developmental stages. The enzymatic treatment is administered locally, and due to rapid metabolism or serum neutralization, its effects are expected to remain confined to the injection site without causing long-term systemic impact. This is corroborated by Olmarker et al. [50], who demonstrated in rabbits that even the most potent proteases cause only localized effects without significant systemic reactions.

## Conclusions

Targeted enzymatic hydrolysis of key ECM components, such as collagen and proteoglycans, offers a promising theoretical avenue for enhancing the plasticity of auricular cartilage. By selectively modulating the mechanical properties of cartilage through enzymes like ChSase and collagenase, this approach may extend the narrow therapeutic window currently limiting non-surgical correction of congenital ear deformities. Among the evaluated enzymes, ChSase appears to be the most efficient in rapidly reducing matrix stiffness, followed by collagenase and hyaluronidase, though each presents distinct mechanistic and safety profiles. While this hypothesis is grounded in established biochemical principles and prior evidence of enzymatic cartilage modulation, its clinical translation will require rigorous validation through future experimental studies. If successful, this strategy could evolve into a minimally invasive, precision-based tool for ear reshaping, ultimately reducing the need for surgical intervention and alleviating psychological burdens for affected infants and their families.
